# Observations on the Practice of Medicine

**Published:** 1807-11

**Authors:** 


### *To the Editors of the Medical and Physical Journal.*

GENTLEMEN,

THROUGH the medium of your publication, I have taken the liberty of offering a few observations on a subject of no small importance to those who have engaged in the important undertaking of practising medicine.

In every situation of life, whether the activity of trade, or the studious concerns of a profession, engage our attention; whether we are hurried in the bustle of a city, or permitted to follow the more tranquil employments of the country, still we have numberless duties to perform, and to perform in such a manner, as that the *means employed*, and the *ends attained*, shall bear alike unsullied, the eye of the strictest enquirer, and the retrospection of our own hearts.

In all these situations, however, there are constantly temptations to deviate from the line of conduct which reason and conscience dictate, suggesting to us easier means of accomplishing our ends, and speedier methods of gaining the laurels we aspire at. At first view, the *medical profession* might seem exempted from such temptations, but, unfortunately, it is exposed to too many; and still more unfortunate is it, that there are so many practicers of it, who strive not to resist them. In a profession so noble in its object, so interesting in its exercise, so admirable in its effects, is it not melancholy to think, that its professors should ever degrade it by practising arts, which would be inexcusable even in the darkest ages of ignorance and doubt; yet that this is not more melancholy than true, I appeal to every candid and disinterested gentleman, who has had any opportunity of witnessing the proprofessional conduct of his medical brethren in a large and populous town.

A Correspondent of yours, in a former Number, has very properly censured "Affectation in Medicine;" he ridicules the sagacious wig, the obedient snuff-box, the knowing stop-watch, the learned phraseology, and the various *tricks* of medical men "*ad captandum*;" but if these little "*ruses de guerre*," in themselves really innocent, be beneath the dignity of the profession, how reprehensible must be the conduct of that man, who endeavours to raise his own reputation, by injuring the fame of another. Yet, how common is such a conduct! Does it not sometimes happen, that a medical man, on being called to attend an individual of a family, whose health is generally intrusted to the care of another, instead of acting upon the principle of "doing as he would be done by," represents the various members of it as having been under hands incompetent to the duties of the profession; says that they look ill, and that a more skilful attendant is necessary for their future health? Or, for instance, a professional gentleman is called in to attend an invalid; he observes a young lady near him, of delicate form and pallid complexion; he requests to feel her pulse; asks her if she ever coughs, has any pain in her side, or experiences difficulty of breathing; and turning to the mother of the family, says, "Madam, your daughter is consumptive: unless immediate measures be had recourse to, she cannot live long." I am aware that in some instances, the most conscientious man might use the language of either of the above instances, (particularly perhaps of the latter, from the increasing prevalence and insiduous nature of consumptive complaints); but before he does, before he alarms the peace of a happy family, let him seriously ask his own heart, whether *his interest*, or *their welfare*, most dictates the interference.

There is another *fashionable trick*, which is of so contemptible a nature, that it cannot be too harshly reprobated; it is that of *making the worst* of every case; of painting the consequences of the complaint in the most glaring colours; of magnifying every danger, and of representing the patient as nearly beyond hope of recovery, while the case is by no means so desperate; and all this merely for the purpose of making the *cure* appear more miraculous. What a sacrifice of principle, for so paltry an end!

When cases of this kind occur, a person who is unacquainted with medicine, and consequently incompetent to judge of the degree of real danger, has, I think, very

 fairly fairly a right to argue that the medical attendant has shewn great ignorance of the nature of the disease, from its termination being so contrary to his prediction; it is true, that unexpected recoveries do *sometimes* take place, but such marvellous examples are by no means so common, as these skilful gentlemen would make us believe. I cannot impress too strongly upon the minds of medical men, the extreme *cruelty* and criminality of sporting in the manner which is so often done with the *feelings* of patients and their friends; this indeed I am convinced is a subject not sufficiently attended to by many, whom no unworthy motives actuate in the exercise of their profession; they are not fully aware how badly an unfavourable prognosis will operate upon a tender and irritable mind, and how much the promise of a *probable recovery* inspires confidence in a patient, and makes both him and his friends bear with cheerfulness and resignation the pains and fatigues of a lingering illness.

I have not sketched an ideal picture; of all that has been stated, I have seen repeated instances, and you, Gentlemen, no doubt, have also seen too many, to allow you to consider these observations incorrect, or irrevelant. The *impolicy* of the conduct I have here deprecated, must be obvious to every reflecting mind. Is it to be supposed that these miserable artifices will always succeed? No, the time must necessarily come, when the *miracles* of these men will be exposed, and a deceived public see the tricks which have been practised upon their ignorance and credulity.

As *Medical Men,* we have the most important duties to discharge, both in relation to ourselves and others; and in order to advance the science of medicine; to uphold the dignity of the profession; to promote the welfare of society, and gain the esteem of our own hearts, we must discharge them as *Men,* as *Gentlemen,* and as *Christians.*

I am, &c. CHIRURGUS.

*Bristol, October* 1807.

